# Extracellular vesicles enhance the *in vivo* antitumor effects of millettia species-derived compounds in chronic myelogenous leukemia therapy

**DOI:** 10.3389/fchem.2024.1425318

**Published:** 2024-07-16

**Authors:** Zongzhou Xie, Xiaozhen Cheng, JianCang Mao, Yingqi Zhu, Le Li, Zhenxin Mei

**Affiliations:** ^1^ Department of Oncology, Haikou City People’s Hospital, Haikou, Hainan, China; ^2^ NHC (National Health Commission of the People’s Republic of China) Key Laboratory of Tropical Disease Control, School of Tropical Medicine, Hainan Medical University, Haikou, Hainan, China; ^3^ Department of Oncology, The Second Affiliated Hospital of Hainan Medical University, Haikou, Hainan, China

**Keywords:** *Millettia species*, *Millettia speciosa* Champ, Homobutein, extracellular vesicles, drug delivery

## Abstract

Several *Millettia species* are being investigated as medicinal ingredients due to their promising anti-cancer and anti-inflammatory properties. However, the application of *Millettia species*-derived compounds has been severely hindered by their poor aqueous solubility, rapid metabolism, and low bioavailability. Extracellular vesicles (EVs), which as membrane-bound phospholipid vesicle initiatively secreted through a variety of mammalian cells, are increasingly recognized as promising drug delivery vehicles. Therefore, EVs are with great potential to enhance both the stability and efficacy of the *Millettia species*-derived compounds in treatment. In this study, extracellular vesicles derived from chronic myelogenous leukemia cells are developed for delivering the extracts of *Millettia speciosa* Champ and *Millettia pachyloba* Drake-derived Homobutein. Notably, Homobutein-loaded EV (hEV) formed a stable and homogenous nanosized particle with high entrapment efficiency up to 55.7%. Moreover, EVs loaded with Homobutein were significantly more potent than free drugs in inhibiting K562 cell proliferation. The results demonstrated that intravenous injection of EV loaded with Homobutein effectively inhibits tumor growth in tumor-bearing mice compared to free Homobutein. Hence, this strategy can effectively enhance the efficacy of *Millettia species*-derived drugs in chronic myelogenous leukemia therapy.

## 1 Introduction

Leguminosae family is widespread in the Hainan, Guangxi, and Guangdong Provinces of China, which roots are used as a traditional medicine by locals for treatment or healthcare practices (Zhang et al., 2020). Numerous *Millettia species* in Leguminosae family have been examined for identifying the biologically active, such as phenylpropanoids, terpenoids, alkaloids, flavonoids, and chalcones. *Millettia speciosa* Champ (Niudali in south China) stands out as one of the most extensively studied species, with its roots serving as a folk medicine of considerable economic and medicinal significance (Zhang et al., 2022; Ajaegbu et al., 2023). Previous pharmacological investigations have demonstrated the efficacy of decoctions containing *Millettia speciosa* Champ in combating tuberculosis, chronic hepatitis, and leukorrhagia (Fu et al., 2016; Huang et al., 2020). *Millettia speciosa* Champ has been recognized for its dual utility as both a culinary herb and a medicinal plant. More than 50 active compounds have been isolated to verify the immunomodulatory, antioxidative, anti-hepatitis, and analgesic activities of the aqueous extracts of *Millettia speciosa* Champ (Yu and Liang, 2019; Huang et al., 2020; Chen et al., 2021; Zhang et al., 2021; Ajaegbu et al., 2023). Consequently, the extraction of *Millettia speciosa* Champ has important application potential and high research value.

Chalcones, compounds with the chemical formula of 1,3-diphenyl-2-propenones, are a cluster of aromatic compounds which represent natural products discovered in numerous medicinal plants (Orlikova et al., 2012). Chalcones as the natural precursors of flavonoids show many bioactivities. Homobutein, a chalcone obtained from another *Millettia species*: *Millettia pachyloba* Drake (Hainan Yadouteng in China), has been reported to possess numerous biological activities such as antitumor, antioxidant, and antiinflammation (Serobatse and Kabanda, 2016; Adeyemi et al., 2019; Yan et al., 2019; Wang et al., 2020). However, the therapeutic effect of Homobutein has not yet been effectively developed because of the poor aqueous solubility, rapid metabolism, and low focus enrichment (Sivakumar et al., 2011).

Extracellular vesicle (EV), a double lipid layer vesicle with uneven size distribution ranging from 50 to 1,000 nm, is secreted from cells through various stimuli and could be taken up by recipient cells (Vader et al., 2014; Pitt et al., 2016). EV has native membrane residing lipids and proteins because it is produced from the outward budding of the plasma membrane, which leads to excellent biocompatibility, low immunogenicity, as well as stability in the blood circulation (Théry et al., 2002; Clayton et al., 2003; György et al., 2015; Ragusa et al., 2015). These excellent features make EV a hopeful carrier for both small molecule and RNA drug delivery (Alvarez-Erviti et al., 2011; Mizrak et al., 2013; Tian et al., 2014). More importantly, recent studies have used tumor cell-derived EV for drugs encapsulation, which facilitates the tumor accumulation of drugs (Tang et al., 2012; Ma et al., 2016). Therefore, using tumor cell-derived EV encapsulating *Millettia species*-derived compounds could be a promising strategy to enhance the antitumor effect.

In this study, we designed a chronic myelogenous leukemia cell-derived EV for the delivery of the extract of *Millettia speciosa* Champ (namely, AeEV) and Homobutein (namelyhEV) to treat chronic myelogenous leukemia. The antitumor effect was verified both *in vitro* and *in vivo*. All of the results indicated that the strategy of EV loading could effectively enhance the therapy effects of the millettia species-derived compounds. This is the first delivery strategy based on naturally extracted ingredients of *Millettia species* and provides a promising strategy for the therapy of chronic myelogenous leukemia.

## 2 Materials and methods

### 2.1 Materials

*Millettia speciosa* Champ root extract was obtained from the National Institutes for Food and Drug Control. Homobutein was purchased from MedChemExpress, Shanghai. 4′,6-diamidino-2-phenylindole (DAPI), LysoTracker Green, dimethyl sulfoxide (DMSO), 3-(4,5-dimethylthiazol-2-yl)-2,5-diphenyltetrazolium bromide (MTT) and other chemicals were obtained from Sigma-Aldrich Chemical Co. (St. Louis, MO, United States). Cell culture plates and round coverslips were purchased from NEST Biotechnology Co., Ltd. (Wuxi, China). Fetal bovine serum (FBS) was from Yeasen Biotechnology (Shanghai) Co., Ltd. Dulbecco’s modified Eagle’s high glucose medium (DMEM) was obtained from Thermo Fisher Scientific (Chicago, IL, United States). BALB/c nude mice were kept in filter-topped cages with standard rodent chow, with water available *ad libitum*, and with a 12 h light/dark cycle. The experimental protocol was approved by Committee on Ethical Animal Experiment at Hainan Medical University (HYLL-2023-182).

### 2.2 EV isolation and characterization

EV was harvested and purified as described previously (Théry et al., 2002; Vader et al., 2014; Pitt et al., 2016). Briefly, K562 cells were seeded in complete growth medium and cells were deprived in FBS before EV isolation. Next, the conditioned medium was collected by centrifugation at 600 g for 30 min to remove cells and debris. Then, the supernatant was centrifuged at 2,000 g for 30 min to clean out apoptotic body. Finally, the EV was collected by centrifugation at 250,000 g for 2 h. The EV pellet was resuspended in PBS and stored at −80°C for further studies. The protein concentration of EV was measured through bicinchoninic acid (BCA) protein assay kit.

The particle size of EV was measured by a Zeta PALS instrument (Brookhaven Instruments, Austin, TX). The morphology of EV was determined through JEOL 100CX II transmission electron microscope (TEM, Tokyo, Japan) and atomic force microscope (AFM, MultiMode 8, Bruker).

### 2.3 Drug loading and entrapment efficiency

The aqueous extract of Niudali and Homobutein were loaded into EV by stirring aqueous extract or Homobutein solution with EV samples (250 μg/mL). The mixtures were vortexed, sonicated for 10 min, and then incubated under magnetic stirring for 2 h at 37°C. The aqueous extract-loaded EV (AeEV) and Homobutein-loaded EV (hEV) were collected through ultracentrifugation at 250,000 g for 2 h. The amount of drug loading and entrapment efficiency (EE) were quantified by spectrophotometer (UV-2600, SHIMADZU, Kyoto, Japan). The absorbance of Niudali aqueous extract was measured at 256 nm and the absorbance of Homobutein was measured at 385 nm. The EE and drug loading were calculated based on the following Equations:

$$\%\text{ EE}=\left(\text{Drug loaded}/\text{Drug added}\right)\times 100$$

Drug Loading=Mass of drug in EV/Mass of EV

### 2.4 *In vitro* cytotoxicity

The cytotoxic effects of EVs on K562 cells were measured by Cell Counting Kit-8 (CCK-8) kit from Dojindo Laboratories (Kumamoto, Japan). The K562 cells were seeded in 96-well plates at a density of 2 × 10^4^ cells per well. Overnight, the cells were treated with various concentrations of empty EV, free aqueous extract, free Homobutein, AeEV, or hEV. After incubation for an additional 24 h, the numbers of viable cells were measured using CCK-8 kits according to the user’s manual. The cell viability of each group was expressed as a percentage of viability relative to that of untreated control cells.

### 2.5 Cellar uptake

To analyze the uptake of EVs *in vitro*. 2 × 10^4^ K562 cells were seeded in each chamber of an imaging dish. The cells were incubated with Cy5-labeled EVs for 4 h, and then stained with Lysotracker and DAPI. After washed by PBS, the cells were imaged under a confocal laser scanning microscopy (CLSM, IX81; Olympus, Tokyo, Japan).

To quantify the uptake of Homobutein, K562 cells were seeded into a 12-well plate (1.5 × 10^5^ cells/well). 24 h later, empty EV, free Homobutein, or hEV were incubated with cells in a serum-free medium. Then, 3 h, 6 h, or 12 h later, cells were treated with RIPA buffer. The concentration of Homobutein was measured by high-performance liquid chromatography.

### 2.6 *In vivo* anti-tumor assay

For the *in vivo* anti-tumor efficacy study, 5 × 10^6^ K562 cells were injected in the front armpit of BALB/c nude mice (female, 5–6 weeks, SPF level, *n* = 6). When tumor volume reached ∼100 mm^3^, different formulations were administrated by tail vein injection (2 mg/kg Homobutein) every 3 days for totally three injections. Tumor sizes were measured every other day and mice were weighed regularly. At 18 days after tumor inoculation, tumors and other tissues were collected and processed for immunohistochemical analysis.

### 2.7 Statistical analysis

Data represent the mean ± SD from indicated independent replicates. Statistical analysis was conducted using GraphPad Prism. For comparisons between two groups, means were compared using the unpaired two-tailed Student’s *t*-test. A value of *p* < 0.05 was considered statistically significant.

## 3 Results and discussion

### 3.1 Construction of the millettia species-derived compounds-loaded EV

**FIGURE 1:**
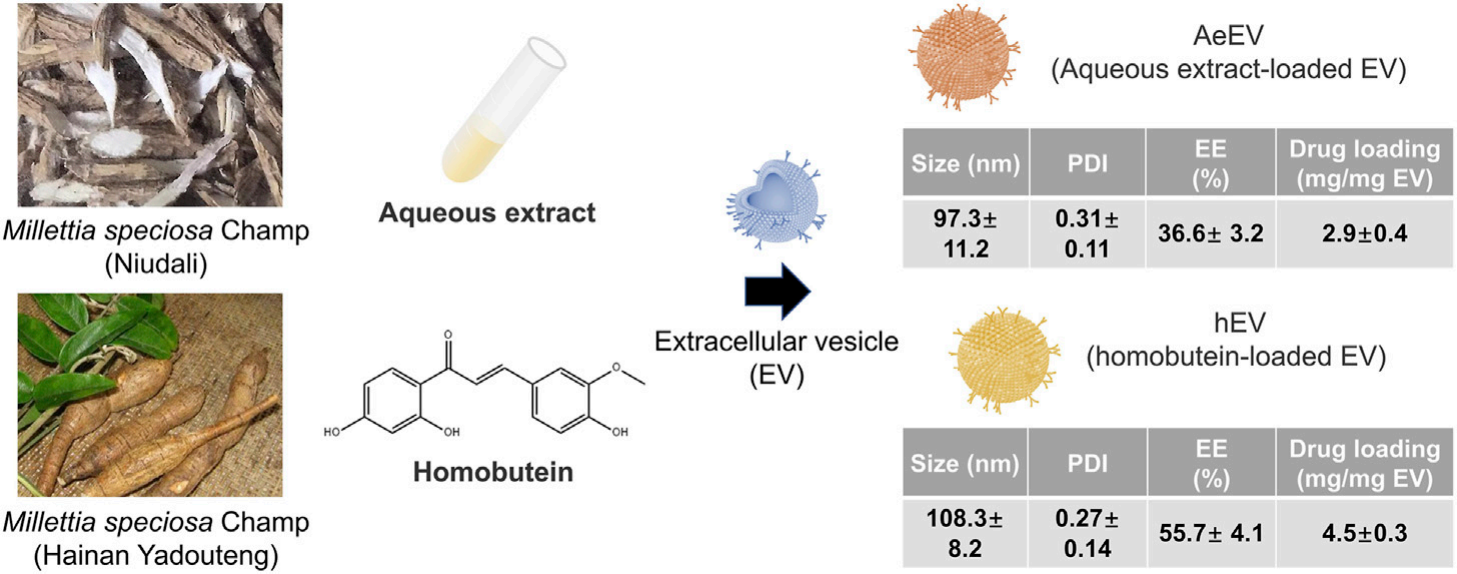
Schematic illustration of the construction of drug-loaded EV. The aqueous extract loaded EV (AeEV) was prepared by mixing aqueous extract with EV. The Homobutein-loaded EV (hEV) was prepared by mixing the chalcone Homobutein from *Millettia pachyloba* Drake with EV. Physicochemical properties of AeEV and hEV was measured. Data are expressed as mean ± SD (*n* = 5).

**FIGURE 2:**
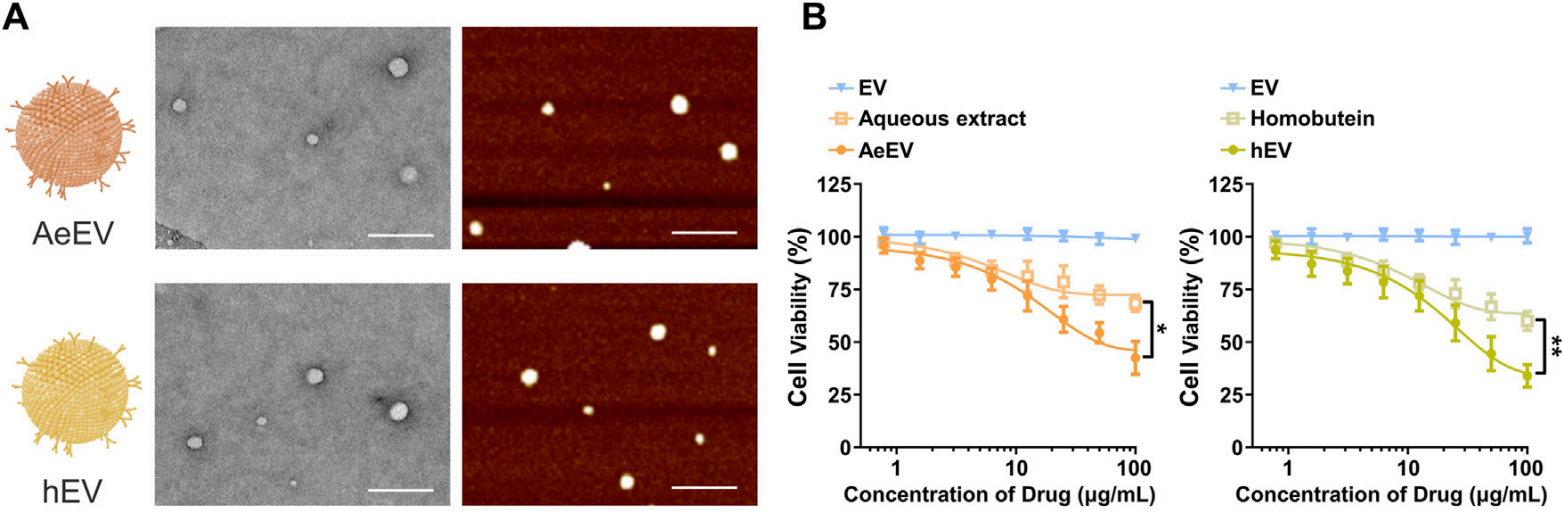
**(A)** TEM and AFM images of the millettia species-derived compounds-loaded AeEV and hEV. Scale bar: 500 nm. **(B)** Cell viability analysis after therapy. The drug concentration was based on aqueous and Homobutein. Data are expressed as mean ± SEM of three independent experiments (*p < 0.01, **p < 0.01).

To verify the feasibility of the *Millettia species*-derived compounds-loaded EV construction. We chose representative tropical Chinese Materia Medica *Millettia speciosa* Champ and *Millettia pachyloba* Drake and utilized its active ingredients for the study (Figure 1). The particle size and polydispersity indexes (PDI) of EV were determined via dynamic light scattering (DLS). The results displayed that the mean particle size of AeEV was 97.3 ± 11.2 nm, and hEV was 108.3 ± 8.2 nm. The PDI of AeEV and hEV were 0.31 and 0.27, respectively, demonstrating a uniform particle size. The results of the transmission electron microscope (TEM) and atomic force microscope (AFM) proved that both AeEV and hEV were spherical in shape with a small size of nearly 100 nm (Figure 2A). The Zeta potential value of EV is −12.8 mV. After the drug loading the more negative Zeta potential value of AeEV (−14.1 mV) and (−15.2 mV) indicates the successful loading of the drugs. Furthermore, the EE and drug loading of AeEV were 36.6% ± 3.2% and 2.9 ± 0.4, respectively. In contrast, the entrapment efficiency (EE) and drug loading of hEV could reach 55.7% ± 4.1% and 4.5 ± 0.3, implying that hEV had relatively higher EE and drug loading efficacy compared with AeEV. Thus, hEV was chosen for further evaluation.

### 3.2 Performance of the EV on cell proliferation

To determine whether AeEV and hEV can be used for the therapy of chronic myelogenous leukemia, *in vitro* cytotoxicity was proceeded by Cell Counting Kit-8 (CCK-8) assay. Both AeEV and hEV led a remarkable cell proliferation suppression in a dose-dependent manner in K562 cells (Figure 2B). The drug-loaded EVs exhibited significantly enhanced anti-tumor activity than free drugs.

### 3.3 Cellular uptake and subcellular localization analysis

**FIGURE 3:**
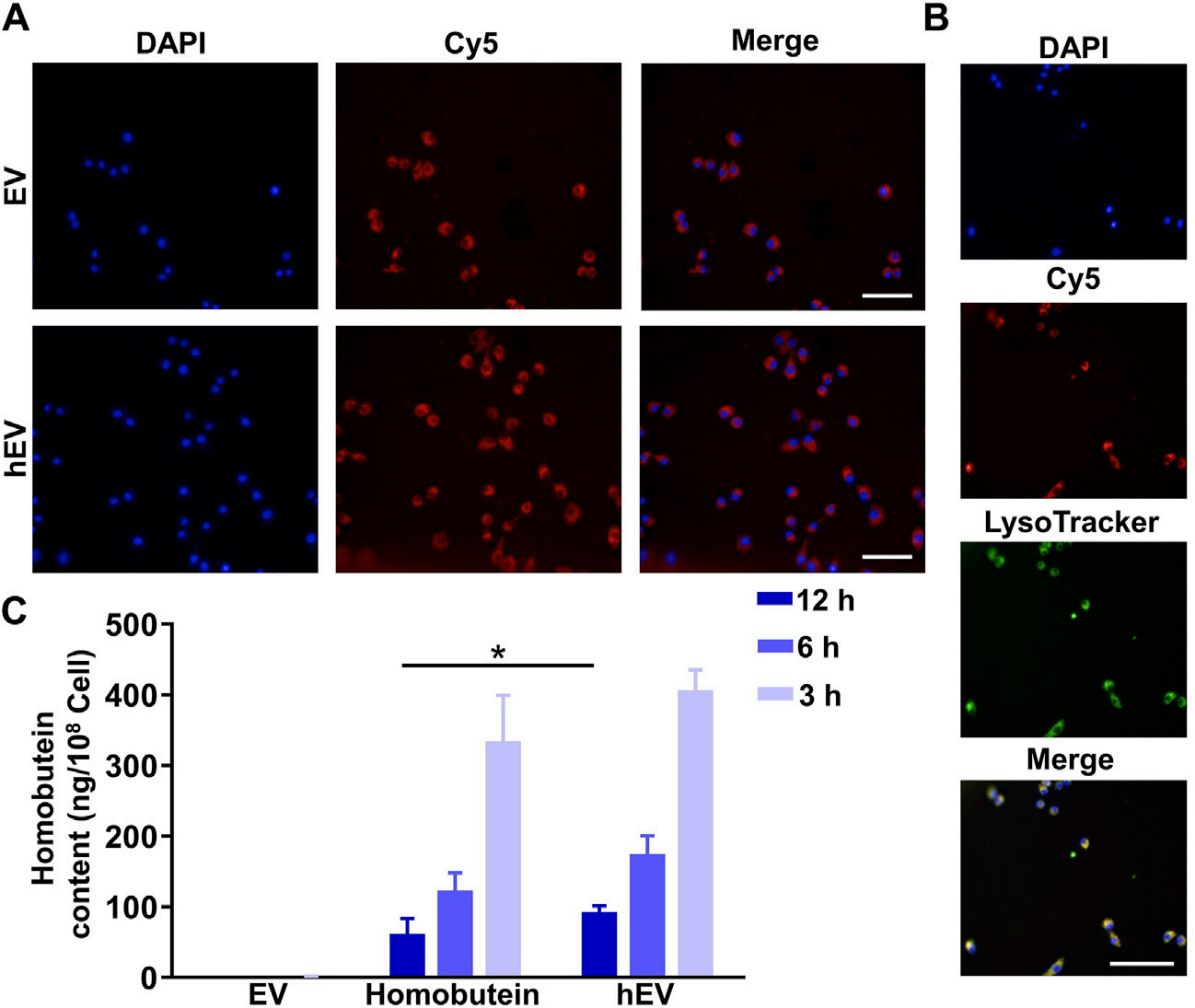
The uptake of hEV. **(A)** Confocal images of K562 treated with fluorescent probe labeled hEV (Cy5-hEV). EV was labeled with Cy5, red; Nucleus was stained with DAPI. Scale bar: 50 μm. **(B)** Confocal images of the subcellular localization of the Cy5-hEV. EV was labeled with Cy5, red; Lysosome was stained with LysoTracker, green; Nucleus was stained with DAPI. Scale bar: 50 μm. **(C)** Homobutein accumulation analysis in K562 at different culture times. The cells were collected and quantified by high-performance liquid chromatography (*p < 0.01).

To test whether hEV could enter into K562 cells, cellular uptake assay was proceeded. K562 cells were treated with Cy5-labeled EV or Cy5-labeled hEV. As shown in Figure 3A, both Cy5-labeled EV and Cy5-labeled hEV were evenly distributed throughout the cytoplasm of the K562 cells. Moreover, to study the lysosome escape of hEV, the lysosome of K562 cells was stained with Lysotracker. The fluorescence of Cy5-labeled hEV was not co-localized with the lysosome, which proved the lysosome escape of hEV (Figure 3B).

To further confirm the uptake of hEV, we then determined the Homobutein accumulation of hEV quantitatively. At 3, 6, and 12 h after incubation, as shown in Figure 3C, the concentrations of Homobutein delivered by hEV were 13.28, 10.47, and 5.73 ng/10^8^ cells on average, respectively. While the concentrations of Homobutein in free Homobutein group were only 2.33, 1.30, and 0.43 ng/10^8^ cells on average, which demonstrated that hEV could increase the uptake of Homobutein.

### 3.4 Tumor inhibitory effect *in vivo*

**FIGURE 4:**
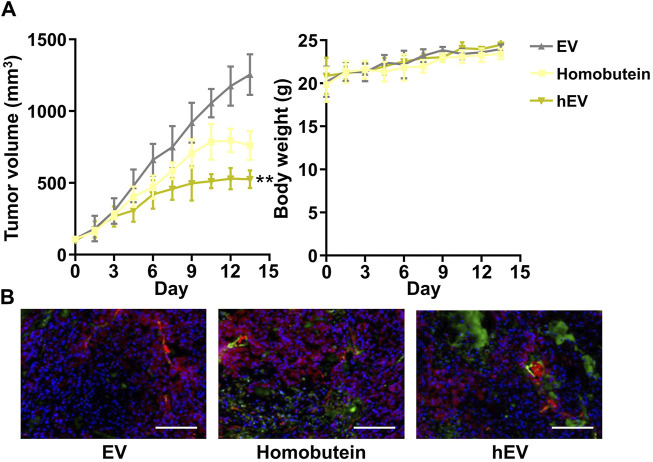
Anti-tumor effect of hEV in xenograft mice model. **(A)** Tumor volume and body weight change of each treatment group. **(B)** Images of immunofluorescence staining of tumor tissues after different treatments. The samples were conducted with TUNEL assays (green fluorescence) and merged with blood vessel signal (red fluorescence). Scale bar: 50 μm (**p < 0.01).

Encouraged by the remarkable cellular uptake and antitumor effect *in vitro* of hEV, we monitored the antitumor therapeutic outcome *in vivo* by a K562 xenografted mouse model. Once the subcutaneous tumor reached 100 mm^3^, three doses of empty EV, free Homobutein, or hEV were administrated via tail vein injection at a 2 mg/mL Homobutein dose every 3 days. At 20 days after tumor inoculation, all the mice were sacrificed, and tumors were acquired for the next step immunohistochemical analysis. As shown in Figure 4A, compared with the empty EV group (1,254 ± 141 mm^3^), the free Homobutein group (760 ± 100 mm^3^) showed smaller tumor size. Moreover, the hEV group had the smallest tumor size (526 ± 61 mm^3^) without body weight reduction. After the treatment, the weight of the extracted tumors and the tumor growth inhibition (TGI) effect were calculated. The average weight of the PBS control is 1.43 ± 0.28 g. The resuced tumor weight was measurged agter the treatment of Homobutein (1.00 ± 0.13 g) and hEV (0.40 ± 0.08 g). The TGI value of hEV (72.3%) is significanly higher than the free drug Homobutein (30.1%). TUNEL assay also demonstrated that the hEV group had the best anti-tumor effect (Figure 4B). Overall, these results indicated that hEV could generate a greater anti-tumor effect *in vivo* without remarkable toxicity.

## 4 Conclusion

In this study, a chronic myelogenous leukemia cell-derived EV was prepared to realize the delivery of the extract of *Millettia speciosa* Champ and Homobutein for treating the chronic myelogenous leukemia. EV, secreted from cells, is a suitable candidate for drug delivery. Specifically, tumor cell-derived EV could lead to the tumor accumulation of drugs. We demonstrated that the entrapment efficiency of Homobutein in hEV could reach as 55.7%. hEV could uptake into chronic myelogenous leukemia cells and realize lysosome escape. Moreover, hEV can lead to treatment outcome both *in vitro* and *in vivo*. Taken together, our prepared hEV provides a tumor cell-derived EV platform for cancer therapy.

## Data Availability

The original contributions presented in the study are included in the article/Supplementary Material; further inquiries can be directed to the corresponding authors.
