# Supplementary material for: Extracellular vesicles enhance the in vivo antitumor effects of millettia species-derived compounds in chronic myelogenous leukemia therapy
Source: Front Chem. 2024 Jul 16;12:1425318. doi: 10.3389/fchem.2024.1425318 (PMC11286385; doi:10.3389/fchem.2024.1425318)
Supplement: Supplementary file 1 [file DataSheet1.docx]

Supplementary Material

Extracellular Vesicles Enhance the *In Vivo* Antitumor Effects of Millettia Species-Derived Compounds in Chronic Myelogenous Leukemia Therapy

**Zongzhou Xie ^1^, Xiaozhen Cheng^1^, JianCang Mao^2^, Yingqi Zhu^2^, Le Li^2*^, Zhenxin Mei ^3*^**

^1^ Department of Oncology, Haikou City People's Hospital, Haikou, Hainan Province, China

^2^ NHC (National Health Commission of the People's Republic of China) Key Laboratory of Tropical Disease Control, School of Tropical Medicine, Hainan Medical University, Haikou, Hainan, 571199, China

^3^ Department of Oncology, The Second Affiliated Hospital of Hainan Medical University, Haikou, Hainan Province, China

*** Correspondence:**Le Li
leli@hainmc.edu.cn

**Zhenxin Mei**

meizhenxin@shhmu.net

# Supplementary Figures


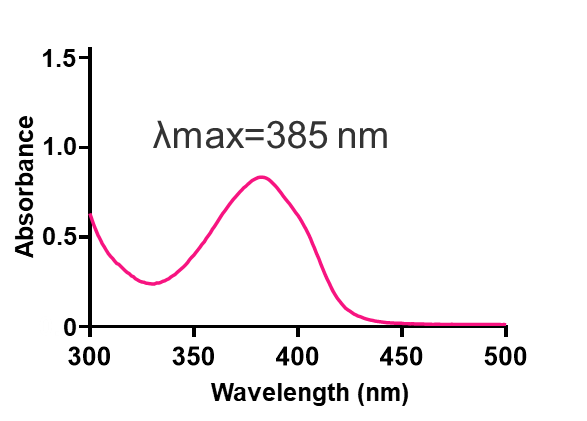


**Supplementary Figure 1.** UV-Vis spectrum of 50 μM homobutein in air-saturated PBS pH 7.4.


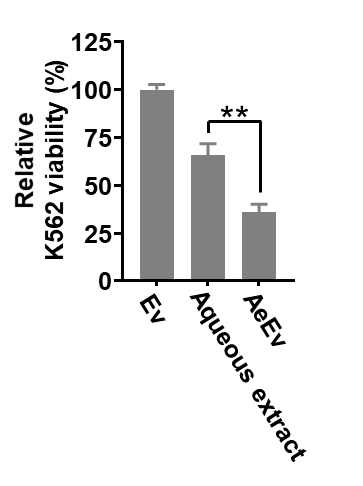


**Supplementary Figure 2.** Cell viability analysis of cells after the treatment with Ev, aqueous ectract, and AeEv. (**p < 0.01).


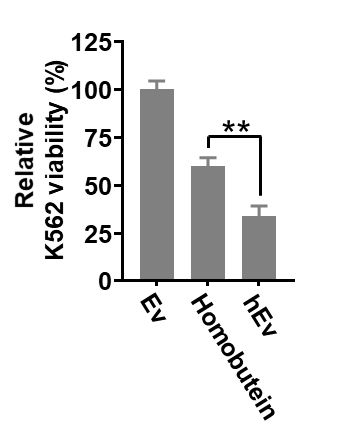


**Supplementary Figure 3.** Cell viability analysis of cells after the treatment with Ev, aqueous ectract, and AeEv. (**p < 0.01).
